# Thoracic Percutaneous Vertebroplasty for the Treatment of Vertebral Hemangioma in a Patient With Forestier’s Disease: A Case Report

**DOI:** 10.7759/cureus.32466

**Published:** 2022-12-13

**Authors:** Renat Nurmukhametov, Brenda Enelis, Edwin Bernard, Manuel de Jesus Encarnacion Ramirez, Medet Dosanov, Juan Sebastian Castro, Ismael Peralta, Yasser Matos Cuevas, Ilya Shirshov, Rossi E Barrientos Castillo

**Affiliations:** 1 Neurosurgery, Peoples’ Friendship University of Russia, Moscow, RUS; 2 Neurosurgery, Central Clinical Hospital of the Russian Academy of Sciences, Moscow, RUS; 3 Division of Spine Surgery, Central Clinical Hospital of the Russian Academy of Sciences, Moscow, RUS; 4 Neurological Surgery, Peoples’ Friendship University of Russia, Moscow, RUS; 5 Vertebrology, Central Clinical Hospital of the Russian Academy of Sciences, Moscow, RUS; 6 Neurosurgery, Dr. Alejandro Cabral Hospital, San Juan, DOM; 7 Neurosurgery, Medical System (MEDSI) Clinical Hospital, Moscow, RUS

**Keywords:** percutaneous vertebroplasty, thoracic forestier’s desease, spinal surgery, vertebroplasty, vertebral hemangioma

## Abstract

Percutaneous vertebroplasty consists of an injection of polymethylmethacrylate in the vertebral body, with the aim of reinforcing the bone structure, preventing vertebral collapse, and achieving analgesic and antitumor effects. It is used in the treatment of patients with aggressive vertebral hemangiomas, as well as compression fractures of traumatic etiology and pathological fractures. Forestier’s disease is also known as senile ankylosing hyperostosis of the spine. It is characterized by hypertrophy of the anterior longitudinal ligament. Depending on the most prominent place of ossification of this ligament, its clinical symptoms vary, with intense pain being the most relevant. Here, we present the case of a 73-year-old female with complaints of intense, constant pain that did not improve with conservative treatment, located at the level of the Th4Th10 vertebrae, radiating along the intercostal spaces, with eight months of evolution with muscular hypertonism. Magnetic resonance imaging of the thoracic spine showed osteochondritis of the thoracic spine and right-sided scoliosis. For hemangioma of the Th6 vertebral body, the patient was referred to the vertebrology department, where she was admitted to undergo percutaneous vertebroplasty of the affected level under fluoroscopic control. In this study, we report the use of percutaneous vertebroplasty as a minimally invasive treatment in a patient with Forestier’s disease, obtaining excellent results, rapid recovery, and minimal hospitalization time, without having to subject the patient to major surgery.

## Introduction

Percutaneous vertebroplasty was described by Galibert et al. in 1987 [1-3]. This consists of injecting polymethylmethacrylate, an acrylic cement, into the vertebral body to reinforce the bone structure, prevent vertebral collapse, and eliminate pain [4-6]. It is frequently used in pathologies such as osteoporotic vertebral fractures, compression fractures, hemangiomas of the vertebral body, myeloma, lymphoma, and osteolytic metastases. Percutaneous vertebroplasty is a minimally invasive treatment that provides the patient with a quick recovery and minimal hospitalization time, obtaining excellent results without the need for major surgery [2,4-6].

In this case, the use of percutaneous vertebroplasty is highlighted in a patient suffering from bone hyperostosis or Forestier’s disease with the presence of hypertrophy and ligamentous flattening, with greater difficulty in recovery and higher risk than in open surgery. In this disease, the ligament that most frequently calcifies is the anterior longitudinal ligament. This pathology is also known as senile ankylosing hyperostosis of the spine. Depending on the most prominent place of ossification of this ligament, its clinical symptoms vary, with intense pain being the most relevant [6-10].

## Case presentation

A 73-year-old woman presented with recurrent, pronounced, and constant pain that did not improve with rest, located at the level of the thoracic spine, radiating pain from Th5 to Th10 spaces on both sides with an evolution of eight months. A physical examination revealed an antalgic position with preservation of the axis of the spine and softened lumbar lordosis. There was no change in sensory reception in the dermatomal Th4- Th10 distribution. On palpation, there was pain along the paravertebral points at the level of the Th4-Th10 vertebrae with an apparent elevated tonus muscular hypertonus. The pain was managed with conservative treatment. However, physiotherapy did not improve her pain.

**Figure 1:**
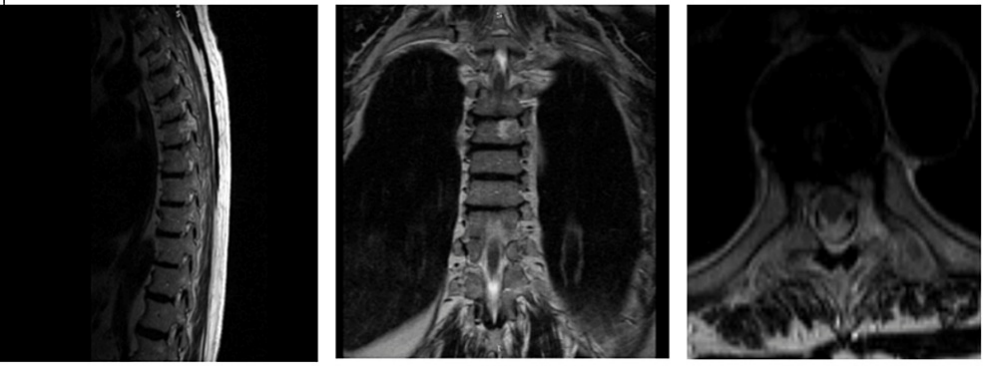
Preoperative surgical stage, from left to right: (A) sagittal, (B) coronal, and (C) axial thoracic magnetic resonance imaging slides on T2. The images show osteochondritis of the thoracic spine, scoliosis on the right side, hemangioma of the Th6 body of the vertebra, and Forestier’s disease.

Magnetic resonance imaging (MRI) of the thoracic spine showed signs of osteochondritis of the thoracic spine, scoliosis where the curve leaned to the right side, a vertebral body hemangioma at the level of Th6 vertebral body, and Forestier’s disease (Figure 1). Therefore, she was referred to the vertebrology department. After admission, percutaneous puncture vertebroplasty of the Th6 vertebra was recommended with bone cement under fluoroscopic control.

Surgical procedure

The patient was placed in a prone-decubitus position. Under intravenous sedation and oxygen therapy, after achieving sepsis and antisepsis with iodinated solutions and fluoroscopic control, marking was performed at the Th6 level. Following this, local anesthesia was infiltrated (novocaine 0.5% 30.0 mL).

**Figure 2:**
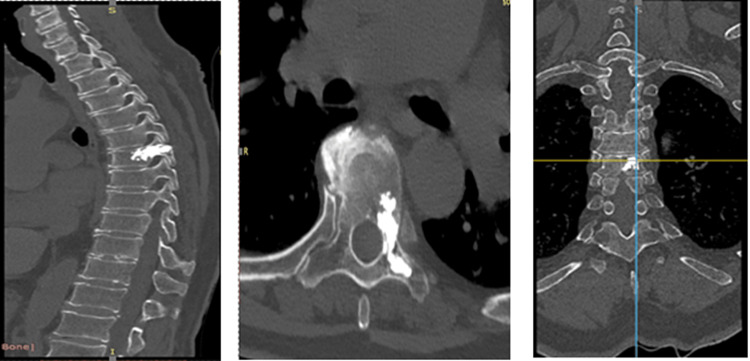
Computed tomography images control: (A) sagittal, (B) axial, and (C) coronal. The images show the placement of the surgical center after the pedicle without apparent effusions.

The Jamshidi needles were placed, locating both pedicles of the Th6 vertebra, and the bilateral transpedicular needles were inserted up to the body of the right-sided vertebra and the left-sided pedicle. Under radiography, in anteroposterior and lateral projections, Stryker-1 PC bone cement was used. The mixing system was started with an additional needle-2 PC, and after four minutes, cement was inserted into the vertebral body. In the left pedicle, about 15 mL of cement in its whole, with everything prepared under fluoroscopic control, no extravasation of cement was detected (Figure 2). Needles were removed, and cutaneous hemostasis was performed by placing sterile dressings.

Results

After treatment, the patient was conscious and on bed rest for two hours. After two hours, she stood up and walked. She did not report pain, and immediate recovery was observed. She was discharged the next day with the place orientations. On follow-up after a week, a control computed tomography (CT) was performed, and then the next control was performed after one month. We obtained an optimal result without subjecting the patient to open surgery.

## Discussion

Bone hemangiomas are usually discovered incidentally in asymptomatic patients. The most frequent locations are the cranial shell and the vertebrae and, to a lesser extent, the long bones (tibia, femur, and humerus). Hemangiomas can be classified histologically into four types, namely, capillary, cavernous, arteriovenous, and venous. The lesions located in the bone are usually of the capillary type [2].

They are usually asymptomatic and discovered incidentally and are most commonly found in the vertebral body, sometimes aggressively spreading, which can cause symptoms. In this case, there was an extension at the pedicle level of the Th6 vertebra, which generated pain in the patient. The association of Forestier’s disease and the marked bone degeneration typical of age led us to perform percutaneous vertebroplasty as the best therapeutic and minimally invasive option, even observing a level of instability. We were able to obtain an excellent result without the need to perform major or open surgery [1,2].

The Russian Association of Neurosurgeons highlights in its therapeutic guide that percutaneous vertebroplasty is a low-cost, minimally invasive technique because it significantly reduces hospitalization time [1].

Imaging methods such as CT and MRI are the gold standard for the detection of aggressive hemangiomas and should be considered in the differential diagnosis in patients with myopathic symptoms and destructive spinal injuries [2,7].

## Conclusions

Vertebroplasty is an excellent option for the treatment of vertebral hemangiomas. It is a minimally invasive method that provides safety and less risk to patients than open or major surgery. In this patient, it generated an excellent result and immediate recovery. Despite the location of the hemangioma and the association of the disease with ligamentous damage, in this case, Forestier’s disease, accompanied by degenerative changes that generated instability, we obtained an optimal result without subjecting the patient to open surgery.
